# Green Seaweed *Caulerpa racemosa* as a Novel Non-Small Cell Lung Cancer Inhibitor in Overcoming Tyrosine Kinase Inhibitor Resistance: An Analysis Employing Network Pharmacology, Molecular Docking, and In Vitro Research

**DOI:** 10.3390/md22060272

**Published:** 2024-06-12

**Authors:** Vincent Lau, Fahrul Nurkolis, Moon Nyeo Park, Didik Setyo Heriyanto, Nurpudji Astuti Taslim, Trina Ekawati Tallei, Happy Kurnia Permatasari, Raymond R. Tjandrawinata, Seungjoon Moon, Bonglee Kim

**Affiliations:** 1 Department of Anatomical Pathology, Faculty of Medicine, Public Health, and Nursing, Universitas Gadjah Mada/Dr. Sardjito General Hospital, Yogyakarta 55281, Indonesia; 2 Department of Biological Sciences, State Islamic University of Sunan Kalijaga (UIN Sunan Kalijaga), Yogyakarta 55281, Indonesia; fahrul.nurkolis.mail@gmail.com; 3 Department of Pathology, College of Korean Medicine, Kyung Hee University, Seoul 02447, Republic of Korea; 4 Korean Medicine-Based Drug Repositioning Cancer Research Center, College of Korean Medicine, Kyung Hee University, Seoul 02447, Republic of Korea; 5 Division of Cardiac, Thoracic, and Vascular Surgery, Department of Surgery, Faculty of Medicine, Public Health, and Nursing/Dr. Sardjito General Hospital, Yogyakarta 55281, Indonesia; 6 Collaboration Research Center for Precision Oncology Based Omics—PKR PrOmics, Yogyakarta 55281, Indonesia; 7 Department of Clinical Nutrition, Faculty of Medicine, Hasanuddin University, Makassar 90245, Indonesia; 8 Department of Biology, Faculty of Mathematics and Natural Sciences, Sam Ratulangi University, Manado 95115, Indonesia; 9 Biochemistry and Biomolecular, Faculty of Medicine, Brawijaya University, Malang 65145, Indonesia; 10 Department of Biotechnology, Faculty of Biotechnology, Atma Jaya Catholic University of Indonesia, Jakarta 12930, Indonesia

**Keywords:** anticancer, green seaweed, natural product, NSCLC, lung cancer, green algae, tyrosine kinase, Caulerpa

## Abstract

The marine environment provides a rich source of distinct creatures containing potentially revolutionary bioactive chemicals. One of these organisms is *Caulerpa racemosa*, a type of green algae known as green seaweed, seagrapes, or green caviar. This organism stands out because it has great promise for use in medicine, especially in the study of cancer. Through the utilization of computational modeling (in silico) and cellular laboratory experiments (in vitro), the chemical components included in the green seaweed *C. racemosa* were effectively analyzed, uncovering its capability to treat non-small cell lung cancer (NSCLC). The study specifically emphasized blocking SRC, STAT3, PIK3CA, MAPK1, EGFR, and JAK1 using molecular docking and in vitro. These proteins play a crucial role in the EGFR Tyrosine Kinase Inhibitor Resistance pathway in NSCLC. The chemical Caulersin (C2) included in *C. racemosa* extract (CRE) has been identified as a potent and effective agent in fighting against non-small cell lung cancer (NSCLC), both in silico and in vitro. CRE and C2 showed a level of inhibition similar to that of osimertinib (positive control/NSCLC drug).

## 1. Introduction

The marine environment is a treasure trove of unique organisms, each harboring potentially groundbreaking bioactive compounds. Among these, the green algae *Caulerpa racemosa*, commonly known as green seaweed, seagrapes, or green caviar, is distinguished by its significant pharmaceutical potential, particularly in the field of cancer research. This species thrives predominantly in regions adorned with coral reefs and enjoys popularity as a dietary staple across Southeast Asian nations [1]. Despite Indonesia’s rich marine biodiversity and vast array of seaweed species, only a select few are harnessed for industrial use [2]. *C. racemosa* is rich in bioactive compounds such as caulerpin, caulersin, caulerchlorin, and racemosins A–C, which confer anti-inflammatory, antioxidative, antibacterial, and antidiabetic properties [3].

This algae’s diverse bioactive components have shown considerable anti-cancer effects, attracting intense interest from the scientific community. *C. racemosa* has been demonstrated to impede cancer cell migration by affecting key proteins in epithelial–mesenchymal transition pathways, a crucial element in cancer metastasis [3]. Additionally, it is renowned for synthesizing caulerpenyne, a compound noted for its antineoplastic and antibacterial activities [4]. A recent study by Kurniawan et al. (2023) further elaborates on its benefits, revealing its antioxidant, anti-inflammatory, and antidiabetic properties, bolstering its candidacy as a functional food or therapeutic agent. The algae’s high carotenoid levels not only amplify its bioactivity but also underscore its value in potential therapeutic applications [5]. These findings suggest that *C. racemosa* holds promise as a natural inhibitor of cancer growth and progression.

Cancer continues to be a major global health challenge, being one of the leading causes of mortality worldwide. In 2021, it was responsible for nearly 10 million deaths, with lung, breast, and colorectal cancers as the primary contributors [6]. Non-small cell lung cancer (NSCLC) has the highest prevalence compared to the other lung cancers, approximately 85% of all lung cancer diagnoses, thereby posing a significant global health challenge [7,8]. As the primary form of lung cancer, the management of NSCLC has significantly advanced with the advancement in targeted therapies and immunotherapies, marking substantial progress in the medical treatment of this disease.

Unfortunately, the treatment of NSCLC is frequently complicated by drug resistance, a critical issue in oncology that significantly diminishes the effectiveness of both traditional and contemporary therapeutic approaches. Resistance to epidermal growth factor receptor (EGFR) inhibitors, often resulting from specific genetic mutations, underscores the challenges in maintaining the success of targeted therapies [9,10]. The development of resistance to osimertinib in NSCLC cells, due to resistant ABCG2 expression, exemplifies the persistent obstacles in treatment efficacy [11]. Similarly, furthermore, although initially effective, immunotherapies such as those involving immune checkpoint inhibitors often see a decline in effectiveness as patients develop resistance over time, complicating long-term management of the disease [12]. Additionally, cisplatin-based chemotherapy, a standard treatment, faces limitations due to inherent or acquired resistance, underscoring the need for ongoing research aimed at overcoming these therapeutic challenges [13].

This persistent issue of drug resistance necessitates a continual evolution in the strategies and approaches for NSCLC treatment, highlighting the importance of innovative research to develop more effective and resilient therapeutic options. Emerging research indicates that *C. racemosa* derivatives exert significant antitumor and antimetastatic effects in experimental models and even induce apoptosis in ovarian cancer cells [14]. Therefore, ongoing research into *C. racemosa* could potentially open new avenues for treating complex diseases such as lung cancer, leveraging its rich array of natural biochemical compounds.

## 2. Results

### 2.1. Compound Identification 

The metabolite profiles of the green seaweed *Caulerpa racemosa* were successfully obtained and analyzed using non-targeted metabolomic profiling with HPLC-ESI-HRMS/MS analysis. Eight potential compounds from *C. racemosa* extract (CRE) have been identified and included as shown in Table 1. 

### 2.2. Pa Value, Toxicity Computation Analysis, Drug Likeness, and Analysis of Network Pharmacology 

We successfully performed Pa value, toxicity computational analysis, drug likeness, and an analysis of network pharmacology on CRE compounds targeting genes and proteins linked to non-small cell lung cancer (NSCLC) in order to determine the pathway that is targeted during the molecular docking stage. The results of these analyses are presented in Table 2. According to the data provided in Table 2, there were five compounds, namely C1, C2, C5, C6, and C7, that showed promising potential in terms of their Pa value for inhibiting kinase activity, a group of enzyme proteins that is responsible in NSCLC. Meanwhile, there were also five compounds, namely C1, C2, C3, C4, and C8, that possessed antineoplastic properties. The following step was determining the LD_50_ values, which were predicted to be more than 1000 and place these compounds in a toxicity class higher than 3. Moreover, each evaluated compound met Lipinski’s Rule of Five, which suggests a good drug likeness; Table 1 noted this information as “Accepted”. These results confirm the therapeutic potential of these CRE compounds in the treatment of NSCLC.

**Figure 1:**
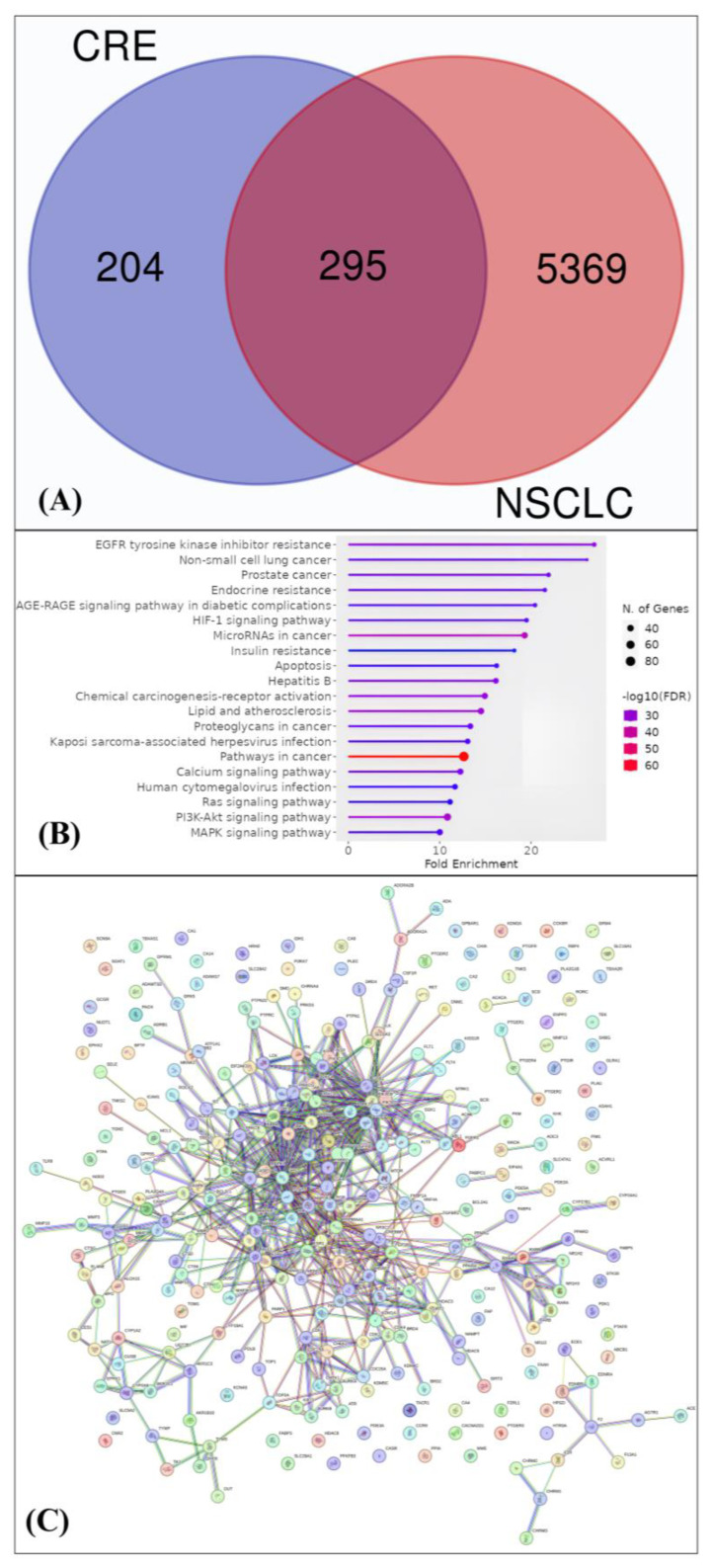
Analysis of network pharmacology of CRE in NSCLC. (**A**) Venn diagram displaying the common targets between CRE and proteins associated with NSCLC. (**B**) Gene ontology biological processes for CRE targets (false discovery rate (FDR) < 0.90). (**C**) Protein-to-protein interaction (PPI) of CRE targets in NSCLC.

A network pharmacology analysis was performed to identify the main proteins involved in the cancer pathway, particularly in NSCLC. The analysis included the listed genes and proteins from disease-related targets and targets derived from the green seaweed CRE. The result was depicted in the Venn diagram (Figure 1A). It was revealed that 295 genes and proteins were in concordance between CRE and NSCLC. Additional analysis of the interactions between target proteins from CRE and their associations with NSCLC revealed several potential targets within cancer pathways, such as resistance to EGFR tyrosine kinase inhibitors, HIF1 Signaling, MAPK, NSCLC, Cancer, PI3K-AKT, and RAS Signaling (Figure 1B). The network pharmacology analysis, as shown in Figure 1B,C, highlights key genes or proteins associated with cancer, including SRC, STAT3, PIK3CA, MAPK (ERK2), EGFR, JAK1, ERBB2 (HER2), MTOR, BRAF, and ALK, as listed in Table 3.

Table 3 shows that CRE has the potential to interact with several target receptors, including SRC, STAT3, PIK3CA, MAPK1, EGFR, JAK1, ERBB2, MTOR, BRAF, and ALK. Notably from the majority of the interactable target receptors, it has the ability to interact with the EGFR Tyrosine Kinase Inhibitor Resistance pathway. Meanwhile, MTOR was found to be linked to PIK3C; BRAF and MAPK1 were found to be linked to EGFR; and ERBB2 and ALK are linked to the common NSCLC and Cancer pathway. These findings suggest that CRE is also implicated in pathways such as PI3K/AKT, HIF-1Alpha, MAPK, and RAS signaling, which are all known to be associated with the promotion of cancer cell growth. Consequently, all ten potential receptors were chosen to proceed with the molecular docking simulation.

### 2.3. Docking Potency of Compounds in the CRE

The molecular docking simulation results targeting specific drug receptors are detailed in Table 4. The table showcases the effectiveness of the identified compounds from CRE, which were utilized for molecular docking with several key proteins: SRC, STAT3, PIK3CA, MAPK1 (ERK2), EGFR, JAK1, ERBB2 (HER2), MTOR, BRAF, and ALK. Osimertinib and mitoxantrone, both targeted chemotherapeutic agents, were used as controls, with their affinity values also displayed in Table 4. The results indicate that Compound C2 consistently exhibits superior binding affinity values compared to the established threshold values for osimertinib and mitoxantrone across all the NSCLC protein receptors. The only exception is protein in PIK3CA, where C2 does not surpass the affinity exhibited by the osimertinib control. These data underscores the potential of Compound C2 as a robust candidate for targeting NSCLC, suggesting its efficacy across a broad spectrum of relevant protein targets. Based on the molecular docking parameters, it is evident that the compound exhibits negative ∆G values similar to the control, albeit with varying degrees. This suggests the presence of synergistic properties between the compounds in the CRE.

We established a threshold for overall ΔG values of −80 for statistical analysis to assess differences between each compound and each control, as detailed in Table 5. Compound C2 demonstrated a significantly higher affinity value compared to both osimertinib and mitoxantrone. Additionally, the affinity values of Compounds C2 and C4 were found to be non-inferior to osimertinib and mitoxantrone. Data supporting these findings, including results from the One-Way ANOVA test, are provided in Supplementary Materials Table S1.

Molecular docking is a computational technique widely used in drug discovery and development to predict the interactions between a small molecule (ligand) and a target macromolecule (receptor). In this study, the binding activity of the substances found in CRE, especially C2, can be assessed through their interactions with various signaling proteins, including SRC, STAT3, PIK3CA, MAPK1, EGFR, JAK1, ERBB2, MTOR, BRAF, and ALK. The assessment is performed by evaluating the substances’ capacity to hinder signal binding to the receptors specified in Table 6. The visual representation of the interaction between amino acids and the compounds identified from other CRE compounds beside C2 and the control against specific receptor proteins can be found in Table S2. The effectiveness of these substances is determined by the strength and amount of amino acid interactions that inhibit signal transmission to the receptors. The flexibility in the use of these substances may be related to the degree of amino acid binding. Additionally, a deeper understanding of a substance’s affinity can be gained by analyzing the strength of the binding interactions, particularly through hydrogen bonds. Most substances present in CRE form hydrogen bonds with amino acids involved in the signaling pathways of EGFR tyrosine kinase. This suggests varying levels of docking activity among the substances, which can be correlated with their chemical structures and functional properties. Furthermore, molecular docking analyses have revealed that Caulerpin or C2 exhibits high binding affinity against specific receptors, indicating its potential as a bioactive compound in combating cancer pathways.

### 2.4. The Potential for Inhibiting NSCLC and the Safety of the CRE

The LD_50_ values were obtained from analyzing CRE compounds on both NSCLC cell lines and normal lung cell lines. Osimertinib and mitoxantrone were used as control samples (Table 7). In NSCLC cells, the LD_50_ values of the CRE samples are classified as “highly toxic” and in normal cells, they are “slightly toxic”. CRE requires a much higher dose compared to both osimertinib and mitoxantrone to effectively kill NSCLC cells. In line with docking simulation, the data indicate that C2 exhibits LD_50_ values on NSCLC cells suggestive of its potent efficacy in targeting NSCLC cell lines. C2 requires a lower dose compared to mitoxantrone and only a slightly higher dose than osimertinib. To NSCLC cell lines, osimertinib was classified as “extremely toxic”, whereas mitoxantrone and C2 were classified as “highly toxic”. In comparison to osimertinib and mitoxantrone, compounds C1 and C4 were classified as “moderately toxic” and required higher dosages to affect NSCLC cell lines. Importantly, whereas the control samples were “moderately toxic” to normal cells, all tested CRE compounds were “slightly toxic” to normal cells. Therefore, CRE compounds could be considered safer to use as alternative treatments for non-small cell lung cancer. Our in vitro study supported the findings from in silico molecular docking studies, which demonstrate that CRE compounds, especially caulersin (C2), have significant potential as anticancer agents for NSCLC, particularly in targeting proteins involved in EGFR Tyrosine Kinase Inhibitor Resistance pathways.

**Figure 2:**
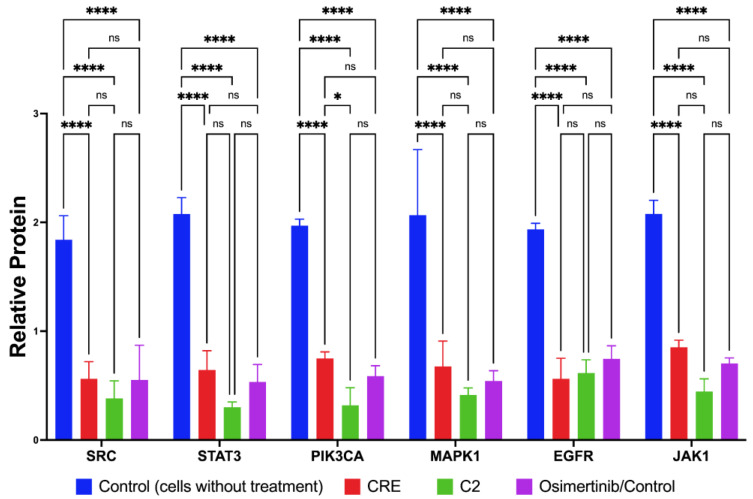
Downregulation of protein-related NSCLC by CRE. ns, not significant (*p* > 0.05); * *p* = 0.0371; **** *p* < 0.0001.

In accordance with our in silico analyses, in vitro studies demonstrated that CRE treatment significantly suppressed the expression level (relative protein expression) of SRC, STAT3, PIK3CA, MAPK1, EGFR, and JAK1 in PC-13 NSCLC cells. Generally, CRE significantly suppressed these proteins’ expression compared to the control without treatment (*p* < 0.0001; Figure 2). Compound C2 also considerably suppressed the expression of these genes in comparison to the control and was significantly more potent in suppressing PIK3CA than CRE itself (*p* = 0.0371; Figure 2). Additionally, the results indicated that both CRE and C2 were non-inferior to osimertinib, a drug used as the treatment control. Because C2 has been shown to have a significantly higher affinity than osimertinib, only C2 (not C1 and C4) was chosen for this analysis (Table 6). These findings conclusively confirm the in silico predictions, highlighting the superior potential of compounds in CRE, particularly C2, in suppressing SRC, STAT3, PIK3CA, MAPK1, EGFR, and JAK1 as proteins involved in the pathways of EGFR tyrosine kinase inhibitors’ resistance in NSCLC.

## 3. Discussion

Research has demonstrated that the extract of *C. racemosa*, the green caviar, can inhibit the migration of HeLa cancer cells by altering the expression of proteins involved in epithelial–mesenchymal transition (EMT) [3]. Additionally, this extract has been shown to induce apoptosis in cancer cells by inhibiting the degradation of the p53 protein, a critical regulator of cell growth and apoptosis [3]. *C. racemosa* is rich in bioactive pigments such as chlorophyll and carotenoids, which are known for their antioxidant, anti-inflammatory, and anti-obesity properties [5]. Furthermore, *C. racemosa* has been studied for its polysaccharide content, which has exhibited antitumor activity [15,16]. This diverse range of bioactive compounds highlights the potential of *C. racemosa* as a source of natural agents for cancer therapy, particularly in targeting pathways underlying cancer cell proliferation and resistance to apoptosis in NSCLC.

**Figure 3:**
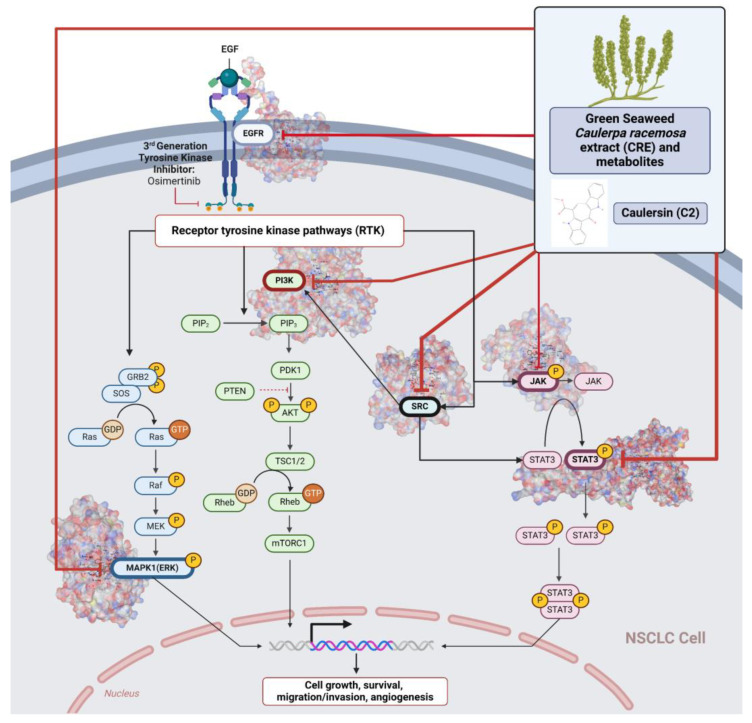
Mechanism of action from CRE in inhibiting NSCLC. Created with BioRender.com Premium License by Fahrul Nurkolis (https://app.biorender.com, accessed on 4 June 2024).

Our study conducted molecular docking analyses on metabolite components of CRE to assess their potential as therapeutic agents. The receptors targeted for binding included SRC, STAT3, PIK3CA, MAPK, EGFR, and JAK1, with osimertinib and mitoxantrone serving as control drugs (Figure 3). Osimertinib, a third-generation EGFR tyrosine kinase inhibitor (TKI), is recognized for its efficacy in treating NSCLC patients harboring EGFR mutations and is preferred as a first-line treatment [17]. It is also globally approved as a second-line treatment for T790M-positive NSCLC patients who experience disease progression during or after EGFR-TKI treatment [18]. Mitoxantrone has been extensively studied for its potential therapeutic applications and its ability to sensitize drug-resistant cancer cells and reverse drug resistance [19]. 

The molecular docking analysis in our study revealed that caulersin (C2), derived from CRE, showed greater overall affinity compared to both osimertinib and mitoxantrone. Caulerpin (C1) exhibited greater overall affinity than mitoxantrone but not osimertinib, while racemosin (C4) demonstrated an overall affinity similar to mitoxantrone. Further analysis indicated that C2 had a significantly higher affinity compared to both osimertinib and mitoxantrone. The affinity values of caulerpin and racemosin were found to be non-inferior to those of the control drugs. Additionally, the in vitro study demonstrated that both CRE and C2 were non-inferior to osimertinib, suggesting that CRE and its components could serve as viable alternatives or adjuncts to existing EGFR tyrosine kinase inhibitors. This non-inferiority is critical as it highlights the potential of CRE and C2 as therapeutic options in cases where there is resistance to traditional EGFR-targeting therapies. This finding underscores the ongoing need for diverse therapeutic strategies in the treatment of advanced NSCLC, which remains a challenging aspect of oncology. The potential of natural products such as CRE in contributing to cancer therapy exemplifies the importance of exploring innovative and alternative approaches to combat drug resistance and improve patient outcomes.

Resistance to EGFR inhibitor therapy presents a significant challenge in the treatment of NSCLC. Initially, the use of EGFR inhibitors such as gefitinib demonstrated promising results in tumor regression and disease stabilization. However, resistance typically develops within 12 to 18 months, often due to secondary mutations within the EGFR, such as the T790M mutation, which diminishes the efficacy of first-generation EGFR inhibitors [20,21]. To combat this, newer EGFR inhibitors like afatinib and osimertinib have been developed to target these resistant mutations, yet resistance remains a major clinical issue, with patients experiencing disease progression within 9 to 14 months [22].

Alternative strategies to overcome resistance have been explored, including combination therapies and the development of other novel drugs. Studies have investigated the efficacy of combining EGFR-TKIs with other agents like TS-1 to combat acquired resistance [23]. The development of fourth-generation targeted therapies, MET inhibitors, antibody–drug conjugates, and immune checkpoint inhibitors have also shown promise in managing drug resistance in NSCLC [9]. Additionally, targeting YAP therapy has been suggested as a potential treatment for NSCLC with acquired resistance to EGFR-TKIs [24].

Further research has identified various mechanisms contributing to resistance, including secondary mutations such as T790M, activation of alternative pathways, phenotypic transformation, and resistance to apoptotic cell death [25,26]. Over half of the patients on first-generation EGFR inhibitors develop resistance through the T790M mutation [27]. Strategies such as combined EGFR/MEK inhibition have been explored to prevent resistance [28]. In light of these findings, the identification of a natural product capable of inhibiting multiple proteins related to EGFR inhibitor resistance offers a potentially transformative approach to addressing this persistent clinical challenge.

Targeting specific proteins such as SRC, STAT3, PIK3CA, MAPK1, EGFR, and JAK1 is crucial for impeding the growth of lung cancer (Figure 3). Inhibition of the Src protein has emerged as a promising therapeutic strategy in lung cancer treatment. Research has demonstrated that Src inhibition can suppress cancer growth through various mechanisms. For instance, Src inhibition has been linked to the induction of FABP4-mediated lipolysis via PPARγ activation, which contributes to the inhibition of lung cancer growth [29]. Additionally, the activation of the Smad2/3 and Src/MAPK pathways, associated with GRP78 signaling in lung cancer, suggests that the knockdown of GRP78 inhibits the activation of Smad2/3 and Src, indicating a causal relationship between GRP78 and these pathways [30]. Moreover, the combined use of kinase inhibitors targeting both the MAPK and Src pathways has shown synergistic effects in suppressing the growth of non-small-cell lung cancer (NSCLC) [31].

Furthermore, Src inhibition has been shown to suppress the migration of NSCLC cells by inhibiting epithelial–mesenchymal transition mediated by STAT3 and Src [32]. Research also highlights the potential of covalent inhibitors targeting the Src kinase to overcome resistance in lung cancer, where elevated Src expression confers resistance to certain anticancer agents [33]. Src has also been identified as a key molecule in conferring resistance to specific treatments in ALK-rearranged lung cancer [34]. Compounds that promote the degradation of mutant EGFR have proven effective in suppressing the growth of lung cancer cells [12]. Disrupting the interaction between RAS and PI3-kinase diminishes the growth and survival of EGFR-mutant lung cancer cells [35]. Furthermore, the inhibition of JAK1 with Anwulignan effectively suppresses the growth of non-small cell lung cancer by targeting the JAK1/STAT3 signaling pathway [36].

With the capability of compounds CRE and C2 to potently inhibit NSCLC cells by targeting these proteins, we have identified a potential “jack of all trades” compound that could inhibit NSCLC cell growth despite further mutations and genetic changes affecting resistance to therapy (Figure 3). Although such potent drugs might be considered “overkill” or potentially “toxic”, our in vitro toxicity tests have shown promising evidence that this is not a concern.

Our study performed cytotoxicity testing to evaluate the safety level of CRE. The value of LD_50_ from the extract was tested on NSCLC cells and normal lung cells, with osimertinib and mitoxantrone as controls. The results showed that the value of LD50 in CRE for the NSCLC cell line is higher than that of the controls, and a significantly greater amount is required to be toxic in normal lung cells. This demonstrates that a higher dosage of CRE is required compared to osimertinib, a proven and frequently used drug to treat NSCLC. Caulersin (C2), in particular, exhibits similar toxicity levels in NSCLC cells, but has a higher threshold for toxicity in normal cells compared with osimertinib. Therefore, both CRE and C2 exhibit relatively high safety levels, making them potential candidates for future chemotherapeutic agent alternatives.

Our research provides a successful investigation that has comprehensively identified CRE compounds that suppress NSCLC cells. This result was accomplished by integrating in silico molecular docking with in vitro validation. By identifying compounds in CRE and understanding their biomolecular actions, we can improve our understanding of potential cancer therapy agents and establish valuable references for future studies, particularly in the field of NSCLC. However, it is important to extend and progress this research to further phases. Studies such as in vivo experiments on animal and clinical trials are necessary to further assess the effectiveness and safety of CRE and compounds in it. This study is currently in the exploratory phase, aiming to determine the presence of the compound in green seaweed extract. Quantitative analysis has not yet been conducted. Consequently, this study is intended to serve as a reference for future research. Therefore, we did not isolate specific compounds but instead used commercially available compounds with higher purity levels. Furthermore, further tests are needed to quantify the presence of the compounds identified in this study to complement current findings. Furthermore, it is essential to separate or to isolate each identified compound in CRE that exhibits anticancer properties using computational analysis. This is necessary to progress its development as a separate product and evaluate its efficacy through in vivo experimentation and subsequent clinical trials.

## 4. Materials and Methods

### 4.1. Caulerpa Racemosa Extract (CRE) Metabolites’ Compound Profiling

The sample collection received approval from both the local authorities and the owner of the green seaweed pond. Green seaweed was obtained from a cultivation pond in Jepara Regency, Central Java Province, Indonesia, located at latitude 6°35′12.5′′ S and longitude 110°38′36.0′′ E. The botanical identification and authentication were verified by an expert biologist and authors. The verification process adhered to the National Center for Biotechnology Information (NCBI) Taxonomy ID 76317 (Eukaryota/Viridiplantae/Chlorophyta/Ulvophyceae/Bryopsidales/Caulerpaceae/Caulepa). Specimens were gathered for future documentation. The researchers assert that all methods employed in this study adhere to the applicable guidelines and regulations for in vitro and algae research. The extract used in this research was obtained from previous researchers [37]. 

The green seaweed *(Caulerpa racemosa*) was meticulously cleansed to eliminate any soil particles, then coiled and desiccated in a Memmert Incubator IN55 oven at 60 °C for 72 h. The dehydrated green seaweed was sliced into small fragments and pulverized into simplicia powder using a blender. The simplicia powder was subsequently extracted using the maceration technique. For maceration, 1000 g of simplicia powder was immersed in 2 L of 96% ethanol for 72 h, with periodic stirring and filtering. The filtrate was concentrated using a rotary evaporator and further evaporated in an oven at 40 °C to obtain a dense extract, which was then stored at 10 °C. This extraction was repeated three times, and the resulting extract was stored at 10 °C. 

An untargeted metabolomics analysis was conducted on green seaweed extracts using both maceration and Soxhlet methods. Liquid Chromatography High-Resolution Mass Spectrometry (LC-HRMS) was utilized for this analysis at the Central Laboratory of Life Sciences, Brawijaya University, Malang, Indonesia. For the analysis, 50 microliters of each extract was diluted with ethanol to a final volume of 1500 microliters, mixed vigorously, and filtered before injection into the LC-HRMS. The LC-HRMS system employed a Thermo Scientific Dionex Ultimate 3000 RSLC nano HPLC system, equipped with specialized solvents and columns, and a Thermo Scientific Q Exactive mass spectrometer for high-resolution full scans and data-dependent MS/MS analysis. This comprehensive procedure ensured the accurate identification of metabolite profiles from the extracts. The methodologies in the particular study also reference earlier research by Nurkolis [38,39]. Each observed compound has not yet been isolated, which is a limitation of our study. In vitro testing will be conducted using commercially available compounds. 

### 4.2. In Silico Study Assessment 

#### 4.2.1. Prediction of Bioactive Compound Activities, Toxicity Analysis, and Drug Likeness

The compounds obtained from green seaweed *C. racemosa* were examined for potential bioactivity using the WAY2DRUG PASS prediction tool (https://www.way2drug.com/PassOnline/predict.php, accessed on 24 February 2024) in order to assess their potential for cancer treatment, especially for treating NSCLC. This analysis involved comparing the input compounds with known compounds that exhibit specific potency, using SAR analysis. The Pa value, or probability of being active, is a prediction score obtained from the web. It indicates the potency of the compound being tested. A Pa value greater than 0.4 suggests that the compound has potential, such as being an anticancer agent, due to its similarity to compounds in the database. The Pa value is used to measure the accuracy of the prediction function. A higher Pa value indicates greater accuracy. In this study, the Pa value is generally limited to values greater than 0.4. Moreover, toxicity and drug likeness analysis encompass a range of pharmacokinetic parameters that play a crucial role in drug development by evaluating the potential harmful effects of a drug. The drug similarity characteristics of each ligand were determined using Lipinski’s Rule of 5 (Ro5). This analysis was conducted using the Protox II database (https://tox-new.charite.de/protox_II/index.php?site=compound_input, accessed on 20 March 2024) and the ADMETLab 2.0 database (https://admetmesh.scbdd.com/service/evaluation/index, accessed on 24 January 2024). The SMILES notation of each compound was used as input for the analysis [40,41,42,43,44,45,46,47]. The SMILES representation for each compound was acquired from PubChem (https://pubchem.ncbi.nlm.nih.gov, accessed on 20 March 2024), and the corresponding data are available in Supplementary Materials Table S3.

#### 4.2.2. Protein Target Identification and Analysis

The SwissTargetPrediction target analysis tool, available at http://www.swisstargetprediction.ch/ (accessed on 24 March 2024), was utilized to conduct target analysis of CRE. The SMILES notation for each compound (as shown in Table S3) and the cut-off score for SwissTargetPrediction were entered. The model’s probability and accuracy threshold were established at 70% (with a range of 0 to 100%) for both Pa values [48]. The genes and proteins associated with NSCLC were obtained from the Genecards Targets database (https://www.genecards.org/, accessed on 24 March 2024). A Venn diagram was employed to delineate the disease-associated targets and targets of CRE, with the purpose of identifying the overlapping targets that are shared by both. The ShinyGO 0.80 tool (http://bioinformatics.sdstate.edu/go, accessed on 24 March 2024) was utilized to carry out the target annotation of CRE. The annotation primarily emphasized biological processes and Kyoto Encyclopedia of Genes and Genomes (KEGG) pathways [49].

#### 4.2.3. Network Pharmacology Analysis

The study includes examining the interactions between specific proteins acquired from CRE and their association with NSCLC using the STRING Database (Search Tool for Retrieval of Interacting Genes/Proteins) Version 12.0 [50]. The input consists of target proteins extracted from CRE and the protein cross-sections linked to NSCLC, as identified through the STRING Database. These include the tyrosine kinase proteins, which are widely recognized for their substantial correlation with the progression of NSCLC. The organism *Homo sapiens* (human) was chosen for this analysis in the STRING Database. In order to guarantee strong and reliable interactions, a threshold of 0.9 was employed as the confidence score cutoff. The data analysis results obtained from the STRING database are presented in TSV (Tab-Separated Values) format. The data are subsequently downloaded and processed utilizing CytoScape Version 3.10.1 for detailed analysis. CytoScape facilitates a thorough examination of network analysis and an exploration of crucial network parameters, including degree, betweenness centrality, and closeness centrality among receptors [51].

#### 4.2.4. Molecular Docking Simulation

The docking simulation used CB-Dock2, an enhanced version of the CB-Dock server, which uses cavity-detection-guided blind docking for protein–ligand blind docking. This method integrates cavity identification, docking, and homologous template fitting. The docking protocol followed the procedures outlined in previous publications [52]. CB-Dock2 is an automated method for docking proteins and ligands. It detects binding sites, calculates their center and size, adjusts the docking box size according to the ligands under investigation, and performs molecular docking using CB-Dock2. CB-Dock simplifies the process of docking and improves accuracy by using the curvature-based cavity detection approach (CurPocket) to anticipate the binding sites of target proteins and CB-Dock2 to determine the binding positions of query ligands. To obtain more extensive information and detailed methodology, go to reference [52]. Furthermore, the receptors with the greatest centrality are chosen for further examination in molecular docking. This consists of receptors that have been observed to be connected to their corresponding signaling pathways. The utilized genes or proteins included the following: SRC, PDB ID: 3F3V; STAT3, PDB ID: 1BG1; PIK3CA, PDB ID: 7R9V; MAPK1, PDB ID: 6G54; EGFR, PDB ID: 1M17; JAK1, PDB ID: 3EYG; ERBB2, PDB ID: 3PP0; MTOR, PDB ID: 4JSV; BRAF, PDB ID: 30G7; and ALK, PDB ID: 5FTO. Prior to docking, the CB2-Dock Server automatically eliminated water molecules and other heteroatoms from the protein structures. All proteins act as receptors or targets for the ligand to dock. The pdb format was acquired from the RSCB Protein Data Bank (https://www.rcsb.org; accessed on 25 March 2024). The ligands were obtained from PubChem in sdf format (https://pubchem.ncbi.nlm.nih.gov; accessed on 24 March 2024). The CB-Dock2 server used in this study is an improved version of the CB-Dock server for protein–ligand blind docking, which integrates cavity detection, docking, and homologous template docking. Given the dimensional structure of a protein and its ligand or target compound, it is possible to predict the binding sites and their affinities for drug discovery with the help of computers. The affinity (∆G) value is used to determine the activity of the ligand or target compound and compare it with a control or standard drug.

### 4.3. In Vitro Study on Cancer Cell Lines

Sigma-Aldrich (Darmstadt, Germany) supplied cell lines for NSCLC cells (PC-9 cell lines Sigma-Aldrich^®^ no. 90071810-CDNA-20UL) and The American Type Culture Collection (ATCC; Manassas, VA, USA) supplied normal lung fibroblast cells (HLF cell lines ATCC^®^ no. PCS-201-013) in the Biochemistry and Biomolecular Laboratory of the Faculty of Medicine Universitas Brawijaya (Malang, Indonesia). PC-9 and HLF (1 × 10^5^) cells were cultured in 96-well plates with Dulbecco’s Modified Eagle Medium (DMEM) supplemented with 10% Fetal Bovine Serum and 1% antibiotics (100 IU/mL Penicillin and 100 µL/mL Streptomycin), following the manufacturer’s protocol. After the cultured cells reach a density of 80%, they are placed in an incubator with a 5% concentration of carbon dioxide at a temperature of 37 °C. Periodically, cells are collected using a trypsin-ethylenediaminetetraacetic acid solution from Thermo Fisher Scientific, Waltham, MA, USA.

#### 4.3.1. Antiproliferative Activity and Cytotoxicity Test of CRE with MTT Assay

The MTT method, as described by Nurkolis et al. in 2023 [38,39], was used to conduct a cytotoxicity test on PC-9 NSCLC cells and HLF normal lung cell lines. Incubate PC-9 and HLF cells on 96-well plates for 24 h. PC-9 and HLF cells were treated with CRE and C2 at concentrations ranging from 0, 35, 70, 105, 140, to 175 µg/mL. Osimertinib and mitoxantrone, which are positive controls obtained from Sigma-Aldrich in Darmstadt, Germany, were subjected to the same treatment as described in previous studies. CRE, C2, osimertinib, and mitoxantrone were introduced and left to incubate for a duration of 24 h. Subsequently, the cells were separated using 1× PBS solution and subjected to incubation with 100 µL of MTT solution at a concentration of 0.5 mg/mL, maintained at a temperature of 37 °C. After a duration of 30 min, a volume of 100 microliters of DMEM stopper reagent was introduced into each well plate. The microplate reader was used to measure the absorbance at a wavelength of 550 nm. In order to mitigate the potential for bias, three sets of triple trials were conducted for each treatment group. The eligible cells are expressed as percentages using the formula provided below:

$$Percentage\;of\;Living\;Cells\;(\%)=\frac{A-B}{C-B}$$

Description = A: cell absorbance with treatment; B: absorbance of blank samples; C: control cell absorbance.

From the results of acute toxicity tests, substances can be classified as follows based on the median lethal dose (LD_50_): less than 5 mg/kg is considered extremely toxic; 5–50 µg/mL is highly toxic; 50–500 µg/mL is moderately toxic; 500–5000 µg/mL is slightly toxic; 5000–15,000 µg/mL is practically non-toxic; and greater than 15,000 µg/mL is relatively harmless [53].

#### 4.3.2. SRC, STAT3, PIK3CA, MAPK1, EGFR, and JAK1 Expression

In vitro analysis of SRC, STAT3, PIK3CA, MAPK, EGFR, and JAK1 expressions was carried out in accordance with the manufacturer’s protocols for the following: Elabscience^®^ Recombinant Human SRC/Proto-oncogene c-Src Protein (His Tag); Elabscience^®^ STAT3 Monoclonal Antibody; Elabscience^®^ PIK3CA Polyclonal Antibody; Elabscience^®^ MAPK kit; Elabscience^®^ Human EGFR (Epidermal Growth Factor Receptor) ELISA Kit; and the Elabscience^®^ Human JAK1 (Janus Kinase 1) ELISA Kit [3]. In order to identify SRC, STAT3, PIK3CA, MAPK, EGFR, and JAK1, the polyvinylidene difluoride membrane was exposed to a blocking solution containing 5% skimmed dry milk in a buffer composed of Tris with Tween (T-TBS) saline buffer. This procedure is carried out in order to inhibit the absorption of any detection reagents by the membrane. The buffer solution consists of 0.1% Tween 20, 20 mmol/L Tris-HCl, 0.138 mol/L Sodium chloride (NaCl; Sigma-Aldrich, Darmstadt, Germany), and has a pH value of 7.4. Alternatively, in order to detect phosphorylated SRC, STAT3, PIK3CA, MAPK, EGFR, and JAK1, a blocking solution containing 5% albumin (specifically bovine serum albumin or BSA) in T-TBS is applied to the membrane. This is conducted to detect the presence of phosphorylated SRC, STAT3, PIK3CA, MAPK, EGFR, and JAK1. 

A specific methodology was employed to evaluate the expression of SRC, STAT3, PIK3CA, MAPK, EGFR, and JAK1. The procedure involves subjecting the cell membrane to primary antibodies, and subsequently binding secondary antibodies conjugated with peroxidase. The primary and secondary antibodies were diluted in a solution that consisted of 5% Bovine Serum Albumin (BSA) dissolved in a T-TBS solution. The study aims to gain insight into the expressions of SRC, STAT3, PIK3CA, MAPK, EGFR, and JAK1 by using a comprehensive technique based on antibodies. This will be achieved by diluting the antibodies appropriately and ensuring the incubation conditions are suitable to maintain precision. In order to provide all the necessary details, the experimental procedure consisted of introducing 5000 PC-9 cells into each well using 100 µL per well. Following a 24 h incubation period, the cells were exposed to CRE dan compounds at a concentration of 18 mM. Subsequently, the acquired data were examined to determine the percentage value in comparison to the control group, which comprised cells that did not receive any treatment or were exposed to 0 mM of CRE. The assessment of percentage (%) value is conducted by utilizing spectrophotometers (SmartSpec Plus from Bio-Rad Laboratories. Inc., Hercules, CA, USA) to measure optical density (OD) at wavelengths of 665 and 620 nm.

### 4.4. Data Management and Analysis

The statistical analysis was performed using GraphPad Prism Premium 10 software on a MacBook computer (GraphPad Software, Inc., San Diego, CA, USA) and SPSS 27.0 for the Windows operating system. The Shapiro–Wilk test was utilized to evaluate the normality of data distributions. If the data exhibited a normal distribution with a significance level of *p* < 0.05, a One-Way ANOVA was employed to compare the differences in means among the treatment groups. If the condition was not met, the Kruskal–Wallis test was utilized. In addition, the LC_50_ (LD_50_, median lethal concentration) was determined for NSCLC cells.

## 5. Conclusions

This research successfully profiled the compounds in green seaweed *C. racemosa* through both in silico and in vitro studies, revealing its potential to combat NSCLC. The study particularly highlighted the inhibition of SRC, STAT3, PIK3CA, MAPK1, EGFR, and JAK1 through molecular docking, which are key proteins involved in the EGFR Tyrosine Kinase Inhibitor Resistance pathway in NSCLC. Furthermore, subsequent in vitro investigations provided new evidence that *C. racemosa* extract (CRE), particularly the compound caulersin (C2), is highly potent in combating NSCLC. This comprehensive combination of approaches identified a promising new source of natural materials for further study and development. Notably, CRE and C2 demonstrated inhibition potency comparable to that of osimertinib. Therefore, future research should involve in vivo and clinical trials to further evaluate the efficacy of CRE and C2, in relation to the doses reported in this study.

## 6. Patents

The extraction method resulting from the work reported in this article has been registered as a patent by Fahrul Nurkolis in Indonesia.

## Figures and Tables

**Table 1:** The metabolites that were detected in green seaweed *C. racemosa* using HPLC-ESI-HRMS/MS analysis.

No	Observed Compounds	Molecular Formula	Observed MW (g/mol)	PubChem ID or Substance ID
C1	Caulerpin	C_24_H_18_N_2_O_4_	398.4	5326018
C2	Caulersin	C_21_H_14_N_2_O_3_	342.3	10593388
C3	Caulerpenyne	C_21_H_26_O_6_	374.4	5311436
C4	Racemosin	C_16_H_16_O_5_	288.29	155148
C5	Hexadecanamide	C_16_H_33_NO	255.44	69421
C6	Oleamide	C_18_H_35_NO	281.5	5283387
C7	Eicosapentaenoic acid	C_20_H_30_O_2_	302.5	5282847
C8	Ageratriol	C_15_H_24_O_3_	252.35	181557

MW: molecular weight.

**Table 2:** The assessment of CRE’s potential as an anticancer agent is performed by examining its structure–activity relationship (SAR) predictions, Pa value, toxicity computational analysis, drug likeness, and analysis of network pharmacology.

No.	Pa Value *		Toxicity Computation Analysis **		Drug Likeness ***		
	Kinase Inhibitor	Antineoplastic	Predicted LD_50_ (mg/kg)	Toxicity Class	Lipinski	Pfizer	GSK
C1	0.727	0.711	1760	4	Accepted	Accepted	Rejected
C2	0.815	0.711	3	5	Accepted	Rejected	Rejected
C3	0.558	0.81	500	4	Accepted	Accepted	Accepted
C4	0.434	0.796	1050	4	Accepted	Rejected	Accepted
C5	0.769	0.393	1000	4	Accepted	Rejected	Rejected
C6	0.838	0.414	750	4	Accepted	Rejected	Rejected
C7	0.828	0.164	10,000	6	Accepted	Rejected	Rejected
C8	0.554	0.945	5000	5	Accepted	Accepted	Accepted

* Way2Drug; ** Protox; *** ADMET.

**Table 3:** Results of the top ten protein-to-protein interaction (PPI) network analyses.

Name	Degree	Betweenness Centrality	Closeness Centrality	Overall Score	Pathway
SRC	33	0.1839	0.4121	33.596	EGFR Tyrosine Kinase Inhibitor Resistance, NSCLC, Cancer
STAT3	33	0.1448	0.4139	33.5586	EGFR Tyrosine Kinase Inhibitor Resistance, HIF1 Signaling, NSCLC
PIK3CA	29	0.0321	0.3569	29.389	EGFR Tyrosine Kinase Inhibitor Resistance, HIF1 Signaling, NSCLC, Cancer, PIK3-AKT, RAS Signaling
MAPK1	26	0.1249	0.398	26.5229	EGFR Tyrosine Kinase Inhibitor Resistance, HIF1 Signaling, MAPK, NSCLC, Cancer, PIK3-AKT, RAS Signaling
EGFR	20	0.0426	0.3767	20.4192	EGFR Tyrosine Kinase Inhibitor Resistance, HIF1 Signaling, NSCLC, Cancer, PIK3-AKT, RAS Signaling
JAK1	16	0.0039	0.3294	16.3333	EGFR Tyrosine Kinase Inhibitor Resistance, NSCLC, Cancer, PIK3-AKT
ERBB2	14	0.0046	0.3569	14.3615	NSCLC, Cancer
MTOR	13	0.0502	0.3582	13.4084	EGFR Tyrosine Kinase Inhibitor Resistance, HIF1 Signaling, Cancer, PIK3-AKT
BRAF	10	0.0095	0.3278	10.3373	EGFR Tyrosine Kinase Inhibitor Resistance, MAPK, NSCLC
ALK	7	0.0002	0.2822	7.2824	NSCLC, Cancer

**Table 4:** The ∆G values of the molecular docking parameter for the identified compounds of CRE.

Compound and Controls as Ligands	SRC	STAT3	PIK3CA	MAPK1	EGFR	JAK1	ERBB2	MTOR	BRAF	ALK	Overall
Osimertinib	−8.8	−7.4	−10.8	−8.3	−8.1	−8.9	−9.4	−9	−8.7	−8.6	−88
Mitoxantrone	−8.2	−7.4	−8.6	−7.8	−7.5	−8.6	−9.1	−8.7	−7.9	−7.8	−81.6
C1	−9.3	−8.3	−9.3	−8.9	−7.3	−7.3	−8.5	−8.6	−9.4	−7.4	−84.3
C2	−9.8	−8.6	−10	−9.4	−9.3	−9.9	−10.6	−10.3	−9.3	−10	−97.2
C3	−8.5	−6.6	−7.7	−7	−7	−7.3	−7.8	−8.1	−8.7	−7.7	−76.4
C4	−8.9	−8.2	−7.8	−7.6	−7.5	−8.3	−7.9	−8.1	−8.3	−8.9	−81.5
C5	−6.4	−5.8	−6.1	−5.2	−5.1	−5.8	−6.7	−6	−6.7	−6.4	−60.2
C6	−6.8	−6.2	−6	−5.7	−5.6	−5.5	−7.4	−6	−6.8	−6.8	−62.8
C7	−7.3	−7.1	−6.8	−6.1	−5.9	−7.5	−8.4	−6.6	−8	−7.7	−71.4
C8	−6.7	−6.2	−8.1	−7.5	−6.1	−6.9	−7.2	−7.3	−7.3	−7.6	−70.9

**Table 5:** The difference between ∆G values of the molecular docking parameter between CRE compounds and control.

Compound	Control			
	Osimertinib		Mitoxantrone	
	Mean Difference	*p*-Value	Mean Difference	*p*-Value
C1	−0.37	0.753	0.27	0.905
C2	0.92	0.036 *	1.57	<0.001 *
C4	−0.65	0.237	0.01	1

* One-Way ANOVA.

**Table 6:** Visual representation of the interaction between amino acids and the compounds identified from CRE against specific receptor proteins.

Proteins	C2
SRC	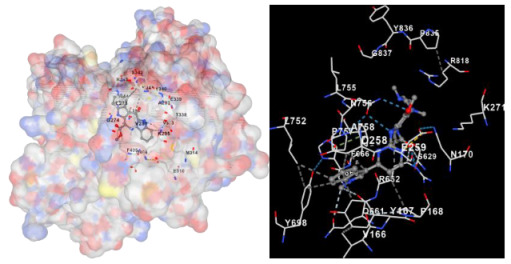
STAT3	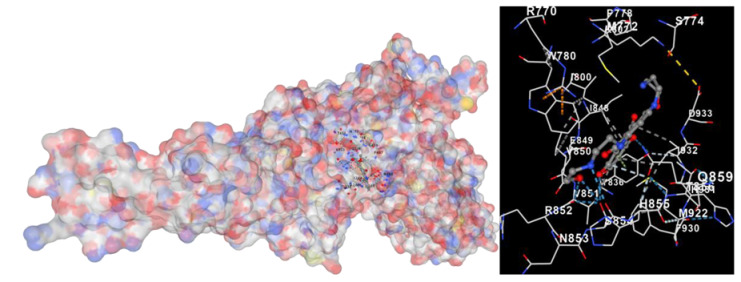
PIK3CA	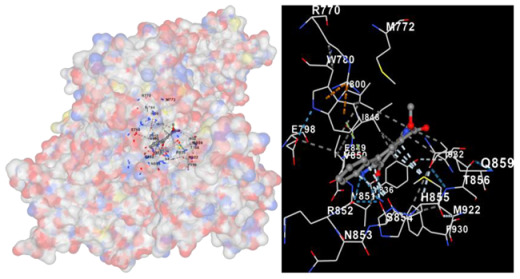
MAPK1	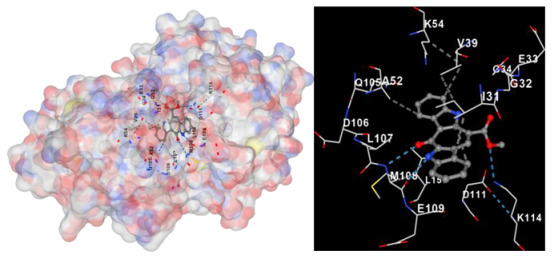
EGFR	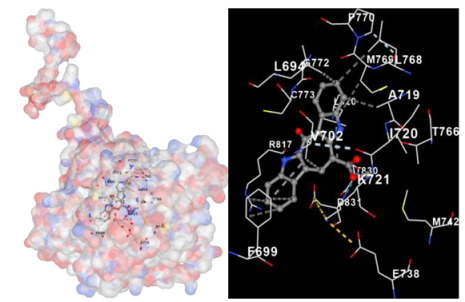
JAK1	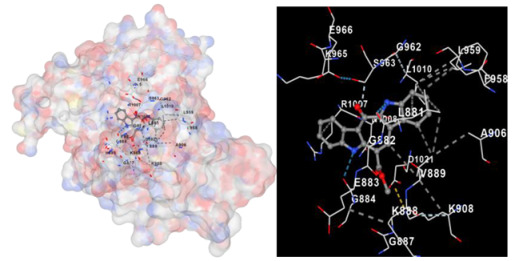

**Table 7:** LD50 values (µg/mL) exhibited by green seaweed *C. racemosa* on NSCLC cell lines and normal cell lines.

Samples	NSCLC Cell Lines (PC-9)	Normal Cell Lines (HLF)
Osimertinib	45.23	340.5
Mitoxantrone	98.4551	454.5505
CRE	289.301	1890.5470
C1	102.4566	913.3324
C2	56.8869	852.5669
C4	183.6501	1090.5321

## Data Availability

The data presented in this study are available on request from the corresponding author.

## Supplementary Materials

The following supporting information can be downloaded at https://www.mdpi.com/article/10.3390/md22060272/s1, Table S1: The analysis of difference between ∆G values of the molecular docking parameter between CRE compounds and control. Table S2: The visual representation of the interaction between amino acids and the compounds identified from CRE against specific receptor proteins. Table S3: The SMILES notation for each compound of green seaweed *Caulerpa racemosa* obtained from PubChem.
