# Supplementary material for: Green Seaweed Caulerpa racemosa as a Novel Non-Small Cell Lung Cancer Inhibitor in Overcoming Tyrosine Kinase Inhibitor Resistance: An Analysis Employing Network Pharmacology, Molecular Docking, and In Vitro Research
Source: Mar Drugs. 2024 Jun 12;22(6):272. doi: 10.3390/md22060272 (PMC11204876; doi:10.3390/md22060272)
Supplement: Supplementary file 1 [file marinedrugs-22-00272-s001.zip › Table S2.pdf]

Compound and Controls as Ligands	SRC	STAT3	PIK3CA	MAPK1	EGFR	JAK1	ERBB2	MTOR	BRAF	ALK
Osimertinib	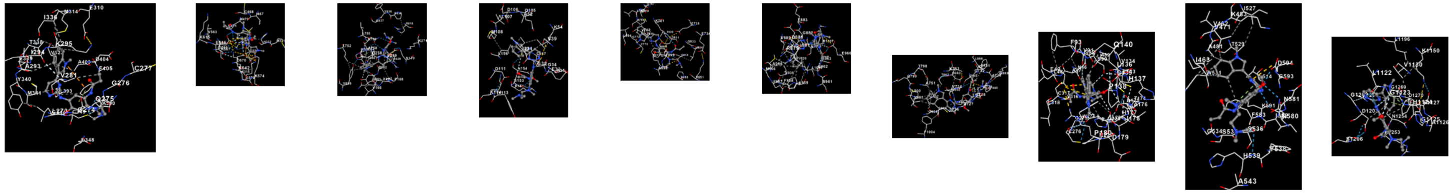									
Mitoxantrone	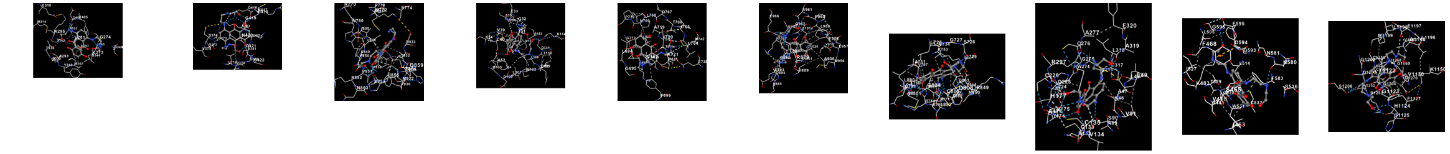									
C1	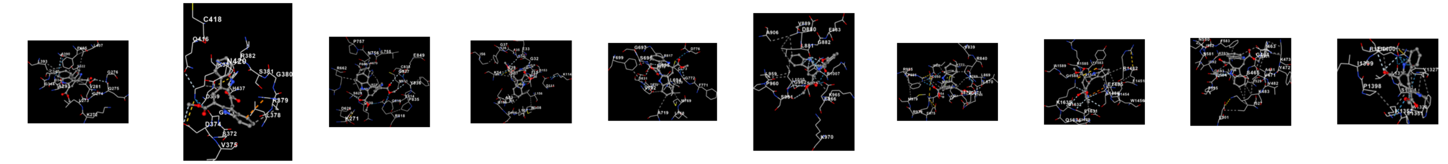									
C2	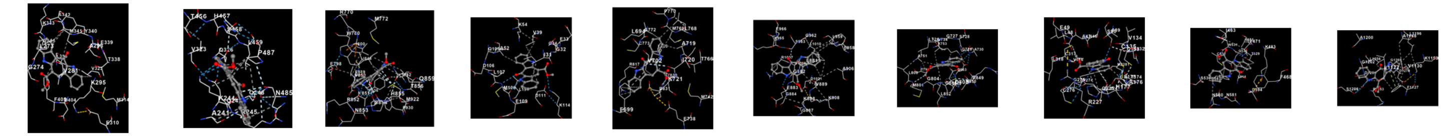									
C4	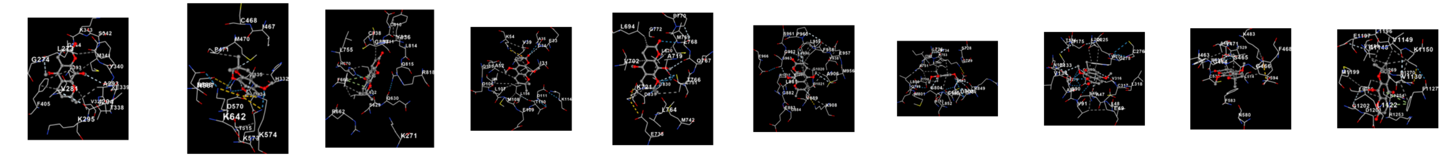									
